# ENDOSCOPIC SLEEVE GASTROPLASTY FOR OBESITY TREATMENT: TWO YEARS OF EXPERIENCE

**DOI:** 10.1590/0102-6720201700010006

**Published:** 2017

**Authors:** Gontrand LOPEZ-NAVA, M P GALVÃO, I BAUTISTA-CASTAÑO, J P FERNANDEZ-CORBELLE, M TRELL, N LOPEZ

**Affiliations:** 1 Bariatric Endoscopy Unit, Madrid Sanchinarro University Hospital, Madrid, Spain

**Keywords:** Obesity. Therapeutics. Endoscopy, Gastrointestinal. Gastrectomy. Minimally Invasive Surgical Procedures.

## Abstract

**Background:**

Bariatric endoscopic techniques are minimally invasive and induce gastric volume reduction to treat obesity. ***Aim***: To evaluate endoscopic sleeve gastroplasty (Apollo method) using a suturing method directed at the greater curvature, as well as the perioperative care, two year safety and weight loss.

**Method:**

Prospective single-center study over 154 patients (108 females) using the endoscopic sleeve gastroplasty procedure under general anesthesia with overnight inpatient observation. Of the154 initial patients, 143 were available for 1-month of follow-up, 133 for 6-month, 64 for 12-month and 28 completed the 24 month assessment. Follow-up was carried out by a multidisciplinary team (nutritionist and psychologist). Outcomes evaluated were: change in BMI; change in body weight (TBWL); % of loss of initial body weight (%TBWL); % of excess body weight loss (%EWL) (segregated in > or <25% and adverse effects. Voluntary oral contrasted radiological examinations were scheduled to assess the gastroplasty at different times post-procedure.

**Results:**

Mean age was 44.9 (23-69) years. At 24 months after the procedure baseline mean BMI change from 38.3 to 30.8 kg/m^2^. TBWL, %TBWL and %EWL were of 21.3 kg, 19.5% and 60.4% respectively. 85.7% of patients achieve the goal of >25% %EWL. There were no mayor adverse events intraprocedure or during the 24 months of follow-up***.***

**Conclusion:**

Endoscopic sleeve gastroplasty with regular monitoring by a multidisciplinary team can be considered an effective, safe and well tolerated procedure for obesity treatment, at least for two years of follow-up.

## RESUMO

**Racional:**

As técnicas de endoscopia bariátrica são minimamente invasivas e induzem à redução do volume gástrico para tratar a obesidade.

**Objetivo:**

Avaliar a gastroplastia sleeve endoscópica (método Apollo) usando um método de sutura direcionado para a grande curvatura, bem como os cuidados perioperatórios, segurança em dois anos e perda de peso.

**Método:**

Estudo prospectivo em um único centro com 154 pacientes (108 mulheres) usando o procedimento endoscópico de gastroplastia sleeve sob anestesia geral com observação do paciente internado durante a noite. Dos 154 pacientes iniciais, 143 estiveram disponíveis para acompanhamento de um mês, 133 durante seis meses, 64 durante 12 meses e 28 completaram a avaliação de 24 meses. O acompanhamento foi realizado por equipe multidisciplinar (nutricionista e psicólogo). Foram avaliados nos resultados as mudanças no IMC, no peso corporal (TWBL), no % de perda do peso corporal inicial (% TBWL) no % de excesso de perda de peso corporal (%EWL) segregado em > ou < 25% e efeitos adversos. Estudos radiológicos contratados orais voluntários foram programados para avaliar a gastroplastia em diferentes tempos após o procedimento.

**Resultados:**

A idade média foi de 44,9 anos (23-69). Aos 24 meses após o procedimento a linha de base média do IMC mudou de 38,3 para 30,8 kg/m^2^. O TBWL, %TBWL e %EWL foram de 21,3 kg, 19,5% e 60,4%, respectivamente, e 85,7% dos pacientes alcançaram o objetivo de >25% EWL. Não houve eventos adversos graves peroperatórios ou durante os 24 meses de acompanhamento.

**Conclusão:**

A gastroplastia sleeve endoscópica com monitoramento regular por equipe multidisciplinar pode ser considerado procedimento eficaz, seguro e bem tolerado para o tratamento de pacientes com obesidade, pelo menos nos dois anos de acompanhamento.

## INTRODUCTION

Many obese cannot sustain sufficient weight loss to improve health with conventional medical lifestyle management (diet, exercise and behavioral therapies)^9^.

Bariatric surgery provides relevant, long-lasting weight loss, and improves obesity-related comorbidities in a relevant percentage of subjects. However, obesity surgery is not exempt from risk, up to 90% of patients reject it, and it is often inaccessible (because of costs, location and lack of resources). Moreover, bariatric surgery is not indicated for some obesity grades. As a result, only a small percentage of the obese population may access bariatric surgery^3^. 

From all the above, less invasive endoscopic procedures are under development for the management of obesity; they provide a higher number of yet untreated obese patients with access to weight loss, allow earlier management, including childhood and juvenile obesity cases, and may be used in the obese elderly^1^ ^,^ ^2^ ^,^ ^4^ ^,^ ^5^. 

Endoscopic sleeve gastroplasty (the Apollo method) is a novel endoscopic technique for the treatment of obesity^8^. 

We report here the effectiveness, safety, weight evolution and up 2-years outcome data from a series of 154 subjects.

## METHODS

### Study population

All procedures followed the Good Clinical Practice guidelines and were performed according to the ethical principles for medical research involving human subjects set forth by the Declaration of Helsinki. The study was approved by the Ethics Committee at Hospital Universitario Madrid Sanchinarro, and registered as 657-GHM. Patients signed a written informed consent.

Similarly, the study was included in ClinicalTrials.gov with ID no. NCT02231970. It uses convenience sampling. Data were prospectively collected for analysis. Patients were selected amongst those visiting the Bariatric Endoscopy Unit at Hospital Universitario Madrid Sanchinarro for obesity between May 2013 and March 2016 who had undergone at least 1 months of multidisciplinary follow-up and who met inclusion criteria.

Inclusion criteria were as follows: obese patients (BMI > 30 kg/m2) who adequately understand and commit themselves to undergo multidisciplinary follow-up for obesity for at least one year.

The technique is contraindicated for the following: acute, potentially bleeding gastric mucosal lesions (ulcers, acute gastritis), neoplastic lesions, hiatus hernia >3 cm, coagulopathy, and psychiatric disorders (the latter assessed using psychological interviews and various blood tests).

### Technique description

The procedure has been descripted previously^6^. The goal of the procedure is to reduce the gastric cavity to resemble a tubular lumen with the greater curvature modified by a line of cinched plications. The technique uses endoscopic transmural suturing throughout the gastric wall to provide a gastric sleeve similar but not identical to sleeve gastrectomy in shape. The gastroplasty uses an endoscopic suture device (OverStitch; Apollo Endosurgery Inc., Austin, Texas, USA) fitted to a dual channel endoscope (GIF-2T160; Olympus Medical Systems Corp., Tokyo, Japan)

The technique is performed under general anesthesia with the patient in the left lateral position, and with endotracheal intubation. An overtube is used for convenience and to increase procedural safety. 

After procedure completion a second endoscopy is carried out to ensure the final tubular configuration is there, to examine any defects requiring complementary closure, and to rule out potential bleeding.

The immediate postoperative period includes inpatient surveillance for 24 h. At 8 h after the procedure liquid tolerance is tested. Blood tests are performed at six and 24 h after the procedure to rule out bleeding. Voluntary oral contrasted radiography is scheduled to assess the gastroplasty at different times post-procedure. 

### Multidisciplinary bariatric team follow-up 

Post-procedure lifestyle intervention includes close follow-up by nutritionist and psychologist weekly or bi-weekly, plus initiating a supervised exercise program. Each individualized program, organized by the team, includes a carefully defined progressive diet, psychological support, physical activity counseling with a planned program, as well as scheduled future visits.

The liquid diet is initiated on the day before the procedure and is continued for at least two weeks after. The patient then progresses from hypocaloric liquids to small semisolid meals over four weeks. An exercise plan that avoids increase in intra-abdominal pressure is recommended during the first month. Initially, walking is encouraged, with a progressive increase in the intensity of exercise as the diet progresses.

### Outcome assessment

The baseline and follow-up examinations included the assessment of weight and height, which were measured using calibrated scales and wall-mounted stadiometer, respectively. For all measurements, patients wore indoor clothing and no shoes. BMI was calculated as weight in kilograms divided by the square of height in meters. The outcomes after 1, 3, 6, 12 and 24 months of follow-up were: 1) change in body weight (TBWL); 2) percentage loss of initial body weight (%TBWL); 3) percentage of excess body weight loss (%EWL) (percentage of weight lost compared with excess weight, defined as current weight minus the weight corresponding to a BMI of 25 kg/m^2^).

The threshold to measure efficacy of endoscopic therapies aimed at weight loss has been suggested by the American Society for Gastrointestinal Endoscopy (ASGE) and the American Society for Metabolic and Bariatric Surgery (ASMBS) taskforce at 25% %EWL at one year^2^. Procedural %EWL outcomes, therefore, were measured against this goal.

**FIGURE 1:**
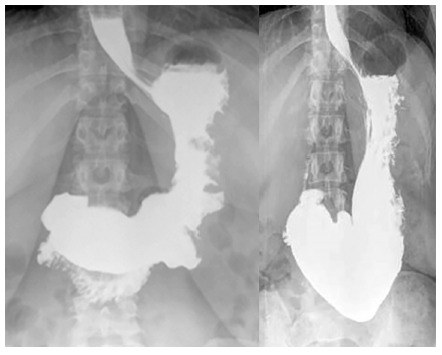
Post-procedure (next day) and 12 month barium radiograph

Figure 1 shows the radiological image at 24 h and one year post-procedure in a 35 years women with a TBWL of 40 kg in one year

### Statistical analysis

This was a prospective pilot study and was therefore carried out without a power calculation. For descriptive purposes, descriptive analysis of study variables was performed using central tendency and dispersion statistics (mean±standard-deviation) for quantitative variables, and frequency and proportion for qualitative ones. The association between changes in body weight parameters and changes in different follow-up groups was also analyzed using the Student t test for pairs. To assess demographic variables (such as age, initial BMI, gender etc.) that may have contributed to better response rates were estimated using the Student's t test for independent samples (for continuous variables) and the χ2 test (for qualitative variables). All p values presented are two-tailed, and statistical significance was defined a priori as p<0.05.Data analyses were performed using SPSS 17.0 (SPSS Inc., Chicago, Illinois, USA).

## RESULTS

The treatment group consisted of 154 patients (108 females and 46 males). The mean age was 44.9+9.5 (23-69) was and the main pre-procedure BMI was 38.3+5.5 (30-47). Of the154 initial patients, 143 were available for 1-month of follow-up, 133 for 6-month, 64 for 12-month and 28 completed the 24 month assessment.

Weight loss results at one month, six months, 12 months and 24 months are shown in Table 1

**TABLE 1:** Weight changes with endoscopic sleeve gastroplasty procedure: results at two years

Variable	Initial (n=154)	1 month (n=143)	6 months (n=133)	12 months (n=64)	24 months (n=28)
Weight (kg)	107.0±19.1	100.1±17.6	90.2±15.9	89.3±18.7	85.9±18.5
BMI (kg/m^2^)	38.3±5.5	35.5± 5.0	32.0± 4.3	31.8± 5.3	30.8± 5.8
TBWL (kg)		8.5±3.9	17.4± 9.2	20.2± 12.2	21.3±13.4
%TBWL (%)		7.7±3.2	15.8± 7.1	18.2± 10.1	19.5±10.5
%EWL (%)		24.8±13.5	47.8±29.4	52.6±31.3	60.4±31.1

All differences between the initial weight and values at 1, 3, 6, 12 and 24 months were statistically significant (p<0.05). There were no mayor intra-procedure adverse events or during the follow up. All patients were discharged on the 1-2 days following the procedure.

Regarding the TBWL at two years of follow-up, 59.1% occurs at first month, 95.2% at six month and 98.7% at first year of follow-up.

In relation with the ASGE goal, 79.7% (n=51) of the 64 patients with one year of follow up reached >25% of EWL. No significant differences were found by gender, initial age or initial BMI.

At two years of follow up 85.7% (n=24) of the patients, obtained >25% of EWL. Groups with better results was younger (mean age 44.7 vs. 50.2, p=0.01). No significant differences were found by gender, or initial BMI.

## DISCUSSION

Early results of the Apollo procedure are promising, demonstrating a 52.6% reduction in %EWL at one year and 60.4% at two year. This result surpasses the mean minimum threshold of 25% EWL as recommended by the ASGE/ASMBS Task Force on Endoscopic Bariatric Therapy^2^.

Most individuals who opt for weight loss procedures have usually struggled for many years with their weight. Endoscopy bariatric techniques, like the Apollo procedure, provides an opportunity to lose weight and help them to change lifestyle habits necessary to perpetuate long-term success. A team of healthcare professionals must be available to provide patients with ongoing education and support. In a recent study^7^, we have publish that during the follow up post-Apollo method, the subgroups with the highest number of nutritional and psychological interactions demonstrated the most favorable weight loss. 

The durability of the endoscopic sleeve gastroplasty at 2-year, along with the weight loss results, suggests that this endoluminal technique remains effective and helpful. It should be noted that no irreversible anatomical alteration occurs in the gastric cavity, the technique is reproducible and repeatable; thus might allow for reintervention in the future to achieve lasting results.

## CONCLUSION

After at least 2-year, the sleeve gastroplasty is an effective, safe, and well-tolerated procedure for the treatment of patients with obesity, with regular monitoring by a multidisciplinary team, a key measure to success.
